# A Randomized Controlled Trial to Evaluate the Impact of an Exercise Therapy Program Based on Sports in People with Acquired Brain Injury: Discover Study Protocol

**DOI:** 10.3390/jcm12227068

**Published:** 2023-11-13

**Authors:** Andrea Gutiérrez-Suárez, Marta Pérez-Rodríguez, Juan José García-Hernández, Beatriz Rodríguez-Romero

**Affiliations:** 1 University of A Coruña, Psychosocial Intervention and Functional Rehabilitation Research Group, Department of Physiotherapy, Medicine and Biomedical Sciences, Faculty of Physiotherapy, Oza, 15071 A Coruña, Spain; 2 AFIPE Research Group, Faculty of Physical Activity and Sports Sciences, Universidad Politécnica de Madrid, 28040 Madrid, Spain; 3 Segunda Parte Foundation, 28034 Madrid, Spain; 4 Faculty of Health Sciences, Exercise and Sport Sciences, University of Francisco de Vitoria, 28223 Pozuelo de Alarcón, Spain

**Keywords:** acquired brain injury, exercise therapy, rehabilitation, sports

## Abstract

Introduction: Acquired brain injury (ABI) constitutes a significant and growing global public health concern. People with ABI often face a range of physical and psychosocial challenges that span the domains of “body structure and function”, “activity”, and “participation”, as defined by the International Classification of Functioning, Disability, and Health. Multidisciplinary approaches based on exercise therapy with social leisure activities are essential to improve physical recovery and health-related quality of life after injury. Methods: Adults with ABI, aged > 18 years, in the subacute or chronic stage (within more than one month after the injury) will be recruited through a rehabilitation center. Adults will be randomized to receive either a racket sports-based exercise therapy program combined with usual care (sET) or usual care alone (UC) using a random number sequence with a 1:1 allocation ratio. sET intervention consists of an 8-week exercise therapy program focusing on different racket sports skills, 1 h in duration, 2 days/week. It will be delivered by a physiotherapist in tailored, face-to-face, group-based sessions. Primary outcomes will be the health-related quality of life (SF-36) and upper extremity motor function (Fugl-Meyer Assessment-Upper Extremity Scale). Discussion: The study proposes an intervention that combines sports-based exercise therapy with usual care. It aims to determine whether this intervention improves the health-related quality of life and upper limb motor function in adults with ABI compared with usual care alone. The results of this study may have clinical implications for the rehabilitation of this population.

## 1. Introduction

Acquired brain injury (ABI) constitutes a significant and growing global public health concern. ABI is a disorder of the mature brain, regardless of its severity or duration, resulting from various etiologies, including stroke, traumatic brain injury, tumors, or infectious diseases [1,2].

People with ABI often face a range of physical and psychosocial challenges, spanning all domains of the International Classification of Functioning, Disability and Health (ICF) model. They experience impairments in the area of “body structure and function” domain (e.g., spasticity, muscle weakness, or poor motor control). In addition, these impairments can have a ripple effect, limiting their abilities within the “activity” domain (e.g., difficulties with fine motor function) and also affecting the “participation” domain (e.g., reduced physical activity and participation in sport) [2]. These patients may experience a reduced physical functioning and health-related quality of life, as well as impaired active participation in community-based physical activity and sports [3,4]. Therefore, the ICF is an important biopsychosocial framework to consider when designing and implementing rehabilitation programs for people with disabilities [5,6]. Hence, evidence suggests that effective rehabilitation interventions for people with ABI should include improvements in all domains [6,7].

Numerous studies have highlighted the positive outcomes of exercise therapy programs for people with ABI. Based on motor learning principles, several systematic reviews have consistently demonstrated the activation of supplementary motor areas and more consistent functional recovery compared to usual care alone [8,9,10,11,12]. In terms of design, multidisciplinary approaches that emphasize the integration of exercise therapy with social leisure activities are considered essential to elicit neuroplastic adaptations that enhance physical recovery [11,13,14,15], and address cognitive and behavioral sequelae following ABI [16,17]. In addition, group-based therapy programs with fewer participants per session have positively affected ABI patients’ quality of life, self-esteem, and social inclusion, as this format encourages and maximizes extensive patient interactions [18,19,20,21].

In terms of context, some studies have proposed exercise programs to ensure a motivational context that facilitates neuroplastic adaptations and behavioral improvements among patients [22,23]. Specifically, an exercise program centered on racket sports is emerging as an accessible therapeutic tool with a strong social component that integrates different body synergies and complex motor strategies, thereby promoting functional performance in both the lower and upper limbs [24].

Despite the noted positive effects of exercise therapy within the ABI population, there remains a noticeable gap in the literature regarding exercise programs that comprehensively address all the domains of the ICF framework. Furthermore, it is frequently observed that these programs do not provide a detailed description of the structure and content of such interventions, thereby limiting their reproducibility [25].

To date, no study has examined the effects of exercise therapy programs based on racket sports for this population. Therefore, the main aim of this study is to design and evaluate the impact of the intervention on health-related quality of life and upper motor control function in ambulant adults with ABI. The secondary aims are to determine the effect of the intervention on functional capacity, mobility, balance, and physical activity participation.

## 2. Materials and Methods

The DISCOVER study protocol was designed in accordance with the Standard Protocol Items: Recommendations for Interventional Trials (SPIRIT) [26] guidelines (Supplementary Table S1). It was registered in the Clinical Trials Registry at clinicaltrials.gov (NCT05358470) and approved by the Regional Ethics Committee for Clinical Research of Madrid (EC 07.22).

### 2.1. Trial Design and Setting

**Figure 1:**
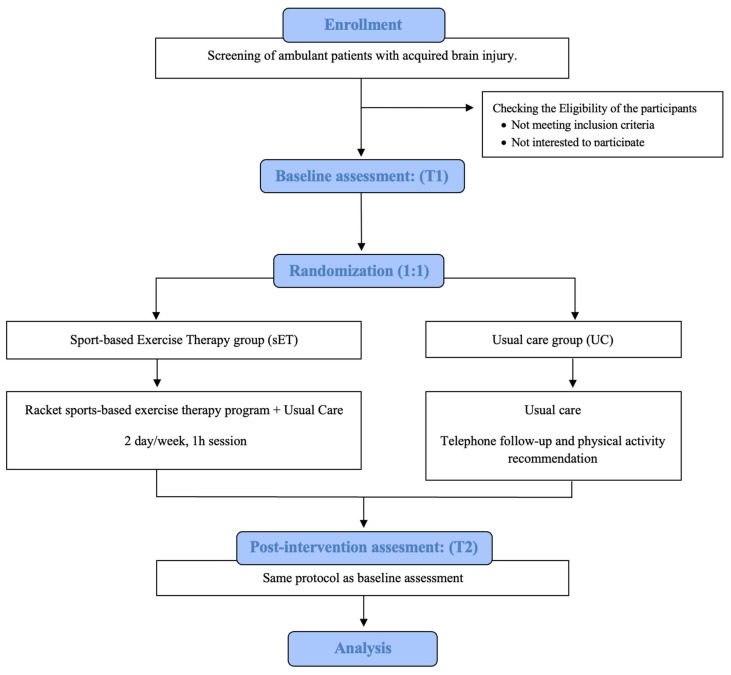
Flowchart of the study design.

The study protocol describes the methods for a single-blind, randomized, controlled trial with two parallel groups. The trial will compare the effectiveness of a sports-based exercise therapy program in combination with usual care (sET group), versus usual care alone (UC group) in ambulatory adults with ABI. A flowchart of the study design according to the CONSORT guidelines is shown in Figure 1.

The study screening, intervention and assessments will be carried out at a rehabilitation center in Madrid, Spain.

### 2.2. Eligibility and Recruitment

The study population consists of outpatients aged 18 years and over with a confirmed diagnosis of subacute or chronic acquire brain injury. A summary of the eligibility criteria is given in Table 1. Recruitment will take place over a period of 3 months to ensure that the target sample size is achieved. Potential patients from the rehabilitation center database will be invited to participate in the study through site visits and telephone calls. Eligibility will be established through prospective face-to-face interviews and eligible individuals will then be enrolled into the study. Throughout this process, participants will provide a signed written informed consent indicating their understanding of the objectives and requirements of the study.

### 2.3. Allocation and Blinding

After completion of the baseline assessment, independent, centralized, stratified block randomization will be used to allocate participants to either the intervention or the control group. Randomization will be performed using a computer-generated random number sequence, with a 1:1 allocation ratio. The assessors involved in eligibility, pre- and post-intervention assessments, and statistical analysis will be blinded to group allocation throughout the entire study period. Due to the nature of the trial, it is not possible to blind intervention parties, including both participant and program staff, to their group assignments.

### 2.4. Interventions

Exercise therapy program based on racket sports (sET):

Participants in the sET group will participate in an exercise therapy program based on racket sports, combined with their usual care sessions as usual. The program will be delivered over sixteen 60 min sessions, delivered twice a week for 8 weeks. The sessions will be delivered in a group format of four to six participants to optimize social engagement rates.

Session content will be carefully designed based on the motor patterns associated with racket sports, allowing for a progressive and effective transition from specific motor function training to broader sports-related tasks. Exercises are continuously tailored and adapted by a physiotherapist in order to meet the individual needs and functional level of each participant, ensuring accuracy and safety. The Template for Intervention Description and Replication (TIDieR) checklist was used to ensure complete reporting of the intervention (Table 2) [27].

Usual care (UC):

Participants in the UC group will continue to receive their usual care for the duration of the study and will be reassessed. Usual care includes a range of therapies (e.g., occupational therapy, physiotherapy, psychological support) provided by the rehabilitation services within the center. After the post-intervention assessment (T2, 2 months after the intervention), participants in this group will have the opportunity to attend the same program sessions if they wish.

### 2.5. Harms

Risk assessment and mitigation strategies will be analyzed and implemented both before and during the trial. Safety and adverse events associated with the groups will be monitored throughout the intervention and reported to the ethics committees if serious. All identified unintended effects or adverse events will be recorded by the research team according to standard guidelines and reported to the ethics committee.

### 2.6. Outcome Measures

#### 2.6.1. Primary Outcome Measures

36-item Short Form Health Survey (SF-36) version 2

This instrument assesses general health in eight domains: physical function, role physical, bodily pain, general health, vitality, social function, role emotional and mental health. Scores above or below 50 (the normative score from the general population) are interpreted as better or worse than the reference population, respectively. This questionnaire has a high validity and test–retest reliability [28].

Fugl-Meyer Upper Extremity Assessment Scale (FM-UE)

The FM-UE is a performance-based index used to assess motor impairment in the upper extremities. It assesses movement of the biceps, triceps, shoulder, elbow, forearm, hand, wrist and finger by performing of 33 tasks. Clinicians rate the patient’s performance on each task for quality of movement on a scale from 0 (no active movement) to 2 (movement appears normal). The maximum score is 66 points, and a high score indicates less impairment. The FM-UE has excellent psychometric properties, including high inter-rater reliability and excellent test–retest reliability and responsiveness [29].

#### 2.6.2. Secondary Outcome Measures

Ten-Meter Walk Test (10MWT) (Gait Velocity)

The 10 MWT is used to assess walking mobility and endurance by measuring walking speed in meters per second over a short distance, and it is calculated as distance divided by time. The patient is instructed to walk at a self-selected speed using any walking aids that may be required. Although there is evidence of excellent internal consistency and test–retest reliability of the test in patients with chronic stroke, there is little evidence of responsiveness in this population. Evidence for validity is also limited but can be inferred from its association with community walking [30].

Six-Minute Walk Test (6MWT) (Gait Endurance)

The 6MWT is used to assess aerobic capacity and endurance. The distance covered in 6 min is used as the outcome to compare changes in performance capacity. It is measured in meters, so the more meters the user is able to cover, the more distance and “normal functioning” will be recorded. The 6MWT has good test–retest reliability in older people and in acute stroke. However, the test has clear face validity in patients with chronic stroke [31].

Timed Up and Go (TUG)

The TUG is used to assess the ability to perform sequential motor tasks related to walking and turning. This test is scored on a scale of seconds measured, with less than 10 s being considered as “functional independence” and more than 30 s being considered “severely abnormal function”. The test–retest reliability of the TUG is high according to previous studies in people with stroke [32].

Berg Balance Scale (BBS)

This scale is a 14-item measure of functional balance, where each item is a five-point ordinal scale ranging from 0 to 4, with 0 representing the lowest level of function and 4 representing the highest level of function. Scores can range from 0 to 56. The higher the score, the better the postural control. The BBS has good internal consistency, confidence interval and test–retest reliability [33].

Global Physical Activity Questionnaire (GPAQ)

This tool consists of 19 questions grouped to capture physical activity across different behavioral domains in which physical activity is performed. It is scored in minutes per day to provide meaningful behavioral units. The GPAQ had low to moderate validity for total PA when compared with PA monitoring questionnaires or accelerometers, and good test–retest reliability [34].

In addition, a physiotherapist will conduct a comprehensive clinical examination to determine whether participants have any physical conditions that may affect their ability to perform certain activities and require adaptations. Participants in both groups will also complete an exercise diary documenting the duration, type and modality (aerobic, strength, flexibility) of their session, which will serve as an additional measure.

#### 2.6.3. Sociodemographic and Anthropometric Data, and Comorbidities

A general screening questionnaire will be used to inquire about possible factors influencing outcomes and to collect baseline information about the sample. Sociodemographic data (date of birth, sex, and school type) and anthropometric data (weight and height to report body mass index (BMI) in kg/m^2^) will be requested, as well as medical diagnoses and comorbid conditions (e.g., controlled epilepsy or asthma medication).

### 2.7. Study Timeline

All participants will attend baseline assessments (post-allocation, T1) prior to randomization into the groups and will be re-assessed at the end of the study period after 8 weeks (close-out, T2). The study schedule is shown in Table 3.

### 2.8. Sample Size

Based on a review of the literature [35,36], people who undergo an exercise therapy program show improvements such as improved quality of life and functionality. Therefore, the sample size was calculated based on the primary outcomes: SF-36 and FM-UE.

Previous studies have shown that a difference of 12.4 points with an SD of 5 points in the FM-UE score is required to detect the minimum clinically important difference between the two study groups [37,38].

Furthermore, Norman et al. conclude that the threshold for detecting changes in health-related quality of life is approximately 5 points [39]. Taking into account the possible number of subjects we could recruit, the sample size is calculated to detect an improvement of 5 points in the summary components of the SF-36 questionnaire with a standard deviation (SD) of 5 points. The SD value was taken as half that of the general population (SD = 10) because we considered our sample to be more homogeneous. To detect this difference between the groups with a 95% confidence interval and 80% power for a bilateral approach, we would need 16 subjects. If we estimated a 15% attrition rate, the final sample size would be 18 subjects in each group.

### 2.9. Data Management and Monitoring

Data will be collected electronically via an encrypted online database tailored to each study procedure, including eligibility, and baseline and post-intervention assessments. No identifying information will be stored in this database. Instead, a unique identification code generated by a random number sequence, will be assigned to each participant in an independent table, ensuring a pseudonymized process. Access to all information will be restricted to a corporate computer and only principal investigators will be authorized to access the data. This process is in place to ensure fidelity of therapy and to ensure that any confidential content is managed in accordance with the protocol. After data entry, a thorough assessment of data consistency will be performed, and any omissions or inconsistencies will be addressed as necessary. Study updates and general results will be communicated to participants via a newsletter.

### 2.10. Statistical Methods

The comparability of the study groups will be assessed by the similarity of the distribution of the variables of interest at baseline. The analysis will follow standard methods for randomized controlled trials, using two-group comparisons for all participants on an intention-to-treat basis. The chi-squared test or Fisher´s exact test will be used to compare proportions. Student´s *t*-test will be used to compare means between groups with normally distributed data. The Mann–Whitney test will be used to compare quantitative variables between groups in the case of a non-normal distribution, as determined by the Kolmogorov–Smirnov test. Correlations between quantitative measures will be determined by Spearman’s rho correlation coefficient. Matched pair data analysis is also calculated. In addition, multivariate analysis will be performed using multiple linear regression and logistic regression, depending on the response under consideration. This will allow for adjustment of the effectiveness of the intervention for potential confounders and the identification of other variables associated with each of the outcomes. Statistical significance will be set at ≤0.05. Data will be analyzed using the Statistical Package for the Social Science (SPSS) software, version 27.0 (IBM Corp, Armonk, New York, NY, USA).

## 3. Discussion

This paper outlines the background and design of a randomized controlled trial that aims to determine the effectiveness of an 8-week exercise therapy program based on sports, combined with usual care, for ambulant adults with ABI, compared to usual care alone.

While previous studies reported positive results from implementing exercise therapy programs centered around sports such as basketball or soccer among people with physical disabilities [40], this study extends the scope of exploration on racket sports, specifically focusing on the ABI population.

A notable strength of this clinical trial lies in the multi-component nature of both the intervention design and assessments: (1) the program encompasses specific neurorehabilitation content alongside sports-based functional exercises that promote interactions and socialization among participants [19,21,40], and (2) the outcome measures involve a range of standardized scales and questionnaires that provide a comprehensive assessment of all the ICF domains [5,6].

As highlighted in earlier research, the proposed sport-based design emphasizes the importance of notably addressing the social domain within ABI rehabilitation, as the focus on the physical domain still predominates in this context. Further studies that integrate sport-based elements into their therapeutic approaches are needed to achieve higher levels of social inclusion within the functional rehabilitation field [9,21,41].

If this program proves effective, it will contribute to clinical practice implications for ABI rehabilitation, offering an evidence-based therapy option that emphasizes the social inclusion perspective and optimally addresses both physical and social recovery. In addition, the results of this study are expected to offer valuable guidance to multidisciplinary teams in achieving optimal levels of health-related quality of life and active lifestyles for their patients.

Finally, it is anticipated that the findings of the trial will be disseminated through peer-reviewed journals and presented at national and international conferences, contributing to broader knowledge and clinical practice in the field of ABI rehabilitation.

## Figures and Tables

**Table 1:** Eligibility criteria.

Inclusion Criteria	Exclusion Criteria
Persons with a medical diagnosis of an acquired brain injury in the subacute or chronic stage (within more than one month of the injury).	Do not have a completed and signed informed consent form.
Be aged >18 years at study entry.	Failure to attend and/or participate in 70% of the program sessions.
To walk independently without the use of assistive devices: score of ≥3, 4 or 5 on the Functional Ambulation Categories (FAC) walking test.	Have medical comorbidities that contraindicate safe exercise (e.g., cardiac or respiratory instability, and uncontrolled seizures).
Be able to understand simple instructions from the exercise therapy program and carry out baseline and post-intervention assessments.	Failure to cooperate during the preliminary tests of the program.

**Table 2:** Template for intervention description and replication checklist for the intervention.

TIDieR Items	Description of Items
Name of the intervention for experimental/comparison group	sET group: racket sports-based exercise therapy program. UC group: usual care
Rationale	Currently, several studies have been carried out that propose a program that combines exercise therapy and adapted physical activity based on different sports (such as basketball or soccer). Nevertheless, no racket sport-based program has been implemented so far. Racket sports emerge as an accessible therapeutic tool with a strong social component that enables the integration of different body synergies and complex motor strategies, thereby promoting functional performance in both the lower and upper limbs. To date, there remains a noticeable gap in the literature regarding exercise programs that comprehensively address all the domains of the International Classification of Functioning, Disability and Health model. This trial raises the need to specifically design exercise therapy programs based on racket sports for this population.
Materials used in the intervention	sET group: The exercise therapy program is centered around different racket sports skills, mainly badminton and tennis, as disciplines which are adapted to participants’ functional condition, as well as different generic equipment such as like balls, chairs, tables, and platforms, among others.
Intervention procedures	sET group: The content of this program is designed to synergize exercise therapy and sports elements. This approach facilitates transitioning from specific motor functional rehabilitation (first eight sessions) to more global skills based on racket sports (final eight sessions). UC group: Usual care encompasses a range of therapies (e.g., occupational therapy, physiotherapy, psychological support) provided by the rehabilitation services within the center. Following the post-intervention assessment, participants from this control group can participate in the same program sessions upon request.
Provider	sET group: The program will be delivered by a physiotherapist with expertise in neurological disorders and adapted physical exercise. UC group: Usual care for this group will be provided by professionals from the rehabilitation center.
Mode of intervention delivery	sET group: Presential, group-based sessions (from four up to six participants) to optimize the rates of social engagement. UC group: Presential, individual sessions.
Setting of intervention	Screening, interventions, and assessments will be conducted at the physiotherapy rooms of a rehabilitation center located in Madrid, Spain.
Dosage	sET group: Participants from this group will receive an 8-week exercise program which consists of sixteen sessions, 1 h session/day for 2 days/week. UC group: This group will continue with their usual care as usual 2 days/week, over 8 weeks and then will be reassessed.
Tailoring	The sessions content will be continuously tailored and adapted by a physiotherapist to meet the individual needs and functional levels of each participant, thereby ensuring precise execution and security.
Modifications	Not applicable.
Fidelity assessment	The therapist responsible for delivering and managing the intervention will conduct a phone follow-up every 2 weeks to monitor the adherence of the sET group to the program. Additionally, this professional will track the progress of participants from the UC group during these follow-up calls.

**Table 3:** Schedule of enrolment, interventions, and assessments from the SPIRIT guidelines.

	Study Period		
	Allocation	Post- Allocation	Completion
TIMEPOINT	T0	T1	T2
*ENROLLMENT:*			
Eligibility screen	X		
Informed Consent	X		
*INTERVENTIONS:*			
Sport-based Exercise Therapy Program + Usual Care		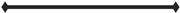	
Usual care		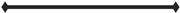	
*ASSESSMENTS:*			
Sociodemographic, anthropometric and comorbidity data		X	
Health-related quality of life (SF36)		X	X
Upper extremity motor function (FM-UE)		X	X
Functional capacity: gait endurance (6MWT)		X	X
Functional capacity: gait velocity (10MWT))		X	X
Mobility (TUG)		X	X
Balance (BBS)		X	X
Physical Activity (GPAQ)		X	X

SF36: Short-Form 36 Questionnaire; FM-UE: Fugl-Meyer Upper Extremity Assessment Scale; 6MWT: Six-Minute Walk Test; 10MWT: Ten-Meter Walk Test; TUG: Timed Up and Go Test; BBS: Balance Berg Scale; GPAQ: Global Physical Activity Questionnaire.

## Data Availability

The datasets are available from the corresponding author on request.

## Supplementary Materials

The following supporting information can be downloaded at https://www.mdpi.com/article/10.3390/jcm12227068/s1. Table S1. SPIRIT 2013 Checklist: Recommended items to address in a clinical trial protocol and related documents.
